# Low-temperature AFM with a microwave cavity optomechanical transducer

**DOI:** 10.3762/bjnano.16.130

**Published:** 2025-10-24

**Authors:** Ermes Scarano, Elisabet K Arvidsson, August K Roos, Erik Holmgren, Riccardo Borgani, Mats O Tholén, David B Haviland

**Affiliations:** 1 Department of Applied Physics, KTH Royal Institute of Technology, Hannes Alfvéns väg 12, SE-114 19 Stockholm, Sweden; 2 Intermodulation Products AB, SE-823 93 Segersta, Sweden

**Keywords:** atomic force microscopy, cavity optomechanics

## Abstract

We demonstrate atomic force microscopy (AFM) imaging with a microcantilever force transducer where an integrated superconducting microwave resonant circuit detects cantilever deflection using the principles of cavity optomechanics. We discuss the detector responsivity and added noise, pointing to its crucial role in the context of force sensitivity. Through analysis of noise measurements we determine the effective temperature of the cantilever eigenmode and we determine the region of detector operation in which the sensor is thermal-noise-limited. Our analysis shows that the force-sensor design is a significant improvement over piezoelectric force sensors commonly used in low-temperature AFM. We discuss the potential for further improvement of the sensor design to achieve optimal detection at the standard quantum limit. We demonstrate AFM operation with surface-tracking feedback in both amplitude-modulation and frequency-modulation modes.

## Introduction

The past two decades have seen the emergence of a variety of remarkable microscopic and mesoscopic optomechanical devices. Through innovative design and fabrication, new record values of fundamental figures of merit have been reported at a surprisingly rapid rate. The field is now mature for the next challenge, that is, application-ready devices. Optomechanical interaction has been proposed to enable or improve many applications [1], including accelerometers [2], tests of quantum gravity [3–5], force microscopy [6–8], magnetometry [9–10], and quantum state transfer [11–13]. In some cases, further improvement on fundamental figures of merit is required, while, in other cases, the difficulty lies in balancing trade-offs to find an optimal design that fulfills the specific requirements of the application. The latter is indeed the case for force sensing in atomic force microscopy (AFM). Force transduction at maximum sensitivity requires detecting the position of a “test mass”, while minimizing the added noise introduced by the detection itself [14–15]. The challenge for high-resolution AFM is designing such a detector for a test mass hosting a sharp tip that is scanned over a surface in ultrahigh vacuum and at ultralow temperature.

In this paper, we report on an AFM cantilever force sensor with an integrated detector consisting of a compact superconducting microwave resonant circuit. Using the principles of cavity optomechanics [16], we detect the deflection of the cantilever from its equilibrium position as a shift of the microwave resonance. We briefly summarize the operating principle of the force sensor based on kinetic-inductive electromechanical coupling (KIMEC), whose design and fabrication were already presented in detail in previous publications by our group [17–18]. Here, we focus on the deployment of the force sensor, demonstrating force-gradient sensing and scanning over a test surface at 10 mK in a closed-cycle dilution refrigerator (DR). We operate the microscope with surface-tracking feedback using the two most common imaging modes of dynamic AFM: amplitude modulation (AM-AFM) and frequency modulation (FM-AFM) [19].

One of the biggest challenges of operating an AFM in a closed-cycle DR is the pulse tube cryogenic head causing significant wideband mechanical vibrations, which can propagate through the cold stages down to the measurement apparatus. However, force sensors with integrated position detectors, as the one presented in this work, do not require free-space optical alignment with a long optical path length. They can therefore be made light and compact, significantly reducing their susceptibility to external vibrations, the complexity of vibration isolation, and their thermal mass. For this prototype, we implement a rather simple vibration isolation which, as we will show, is sufficient to demonstrate imaging features in a 1 µm scan field, but has not been tested to atomic resolution.

## Results and Discussion

### Force sensitivity and imaging

The microcantilever in dynamic AFM operates as a resonant force transducer. Each individual eigenmode is described by a mechanical susceptibility χ, expressing cantilever deflection ζ in response to a force *F* at the frequency Ω:


[1] $$\chi \left(\Omega \right)=\frac{\zeta \left(\Omega \right)}{F\left(\Omega \right)}=\frac{1}{m_{\text{eff}}\left(\Omega_{\text{m}}^{2}-\Omega^{2}+i\Omega \Gamma \right)},$$

where 
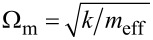
 is the resonance frequency and Γ = η/*m*_eff_ the damping rate, with *m*_eff_, *k* and η being, respectively, effective mass, stiffness, and damping coefficient of the eigenmode.

A key figure of merit of a force transducer is its force sensitivity determined by all sources of noise. The total noise can be expressed as the sum of the power spectral densities (PSDs) of uncorrelated contributions such as the thermal fluctuations in cantilever deflection 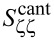
(Ω) [m^2^/Hz] at the effective mode temperature *T*, measurement back action noise 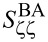, and the frequency-independent added noise of the detection *S*_VV_ [V^2^/Hz], which is converted to an equivalent deflection noise 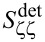 via the detector responsivity α [V/m]:


[2] $$\begin{array}{c} S_{\zeta \zeta }^{\text{tot}}\left(\Omega \right)=S_{\zeta \zeta }^{\text{det}}+S_{\zeta \zeta }^{\text{cant}}\left(\Omega \right)\\ =\frac{S_{\text{VV}}}{\alpha^{2}}+S_{\zeta \zeta }^{\text{BA}}\left(\Omega \right)+2k_{\text{B}}T\eta {\left|\chi \left(\Omega \right)\right|}^{2}. \end{array}$$

Neglecting back action, the noise-equivalent force for a given measurement bandwidth Δ*f* is given by


[3] $$F_{\text{N}}\left(\Omega \right)={\left(\int_{\Omega -\pi \Delta f}^{\Omega +\pi \Delta f}\frac{S_{\zeta \zeta }^{\text{tot}}\left(\Omega \right)}{{\left|\chi \left(\Omega \right)\right|}^{2}}\frac{\text{d}\Omega }{2\pi }\right)}^{1/2}.$$

This quantity is minimized at the mechanical resonance frequency, where |χ(Ω_m_)|^2^ = *m*_eff_/*k*η^2^, resulting in a minimum detectable force,


[4] $$F_{\text{min}}=F_{\text{N}}\left(\Omega \right)\approx \sqrt{\left(\frac{S_{\text{VV}}k\eta^{2}}{\alpha^{2}m_{\text{eff}}}+2k_{\text{B}}T\eta \right)\Delta f}.$$

It is worth noting that Equation 4 clarifies a recurring misconception. Decreasing stiffness *k* does not necessarily reduce *F*_min_. Rather, it relaxes the constraint on the detector’s added noise for the force sensor to operate in the thermally limited regime 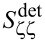 ≪ 
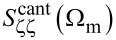
, where *F*_min_ ≃ 
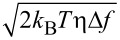
. Achieving this limit can be especially challenging at cryogenic temperatures.

Heritier et al. achieved a record force sensitivity of 0.16 aN/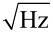 using a Si nanoladder with Ω_m_/2π = 5.5 kHz and stiffness *k* = 6.5 µN/m at *T* = 100 mK [20]. AFM usually requires stiffness values of the order of 100 N/m to avoid the tip jumping to contact and sticking to the surface [21]. Such a stiffness would necessitate seven orders of magnitude improvement in detector noise to achieve an equivalent force sensitivity. Reduction of detection noise is therefore critical for improving low-temperature AFM.

### AFM sensor and detection principle

**Figure 1:**
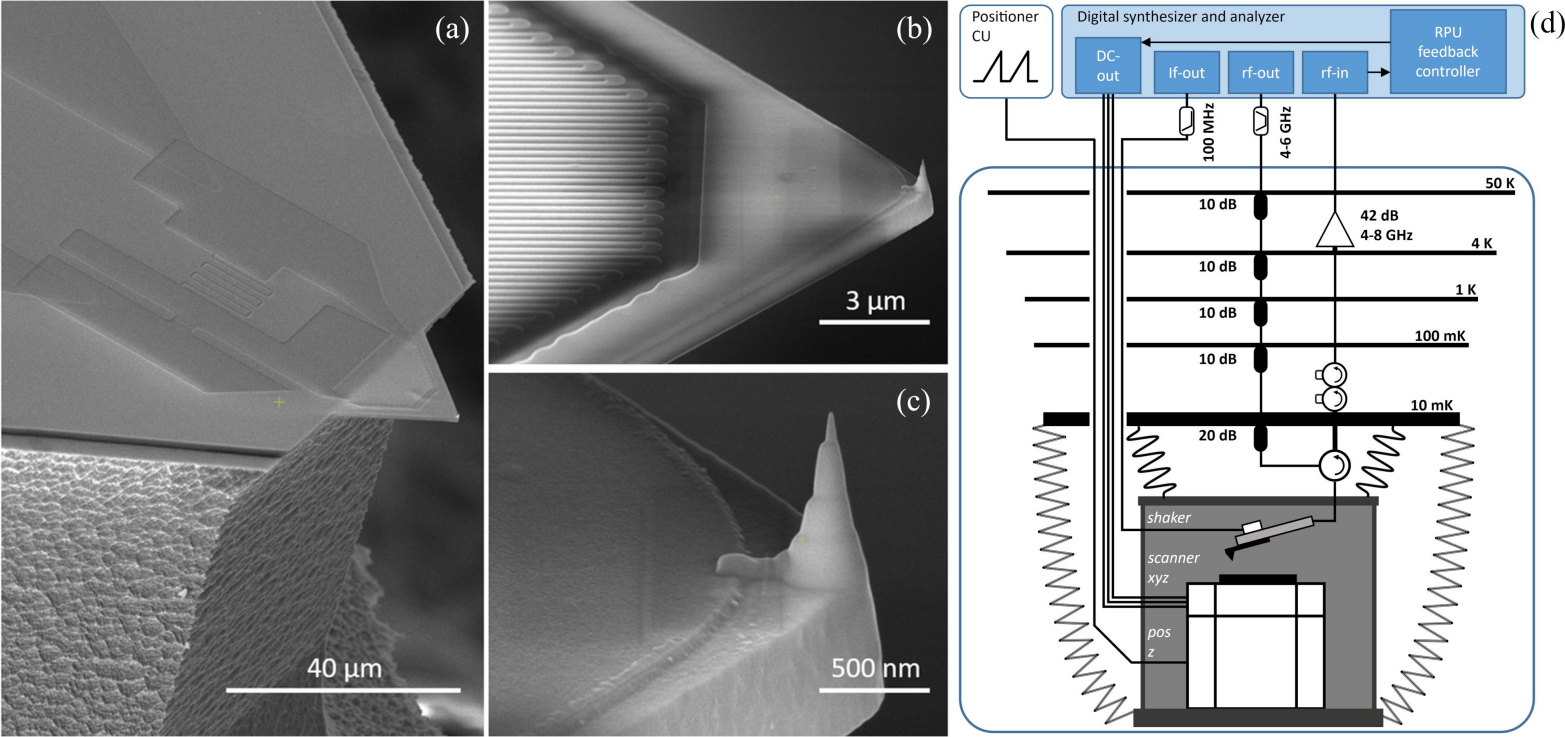
(a–c) Scanning electron micrograph of the probe, featuring a Si–N triangular cantilever released from the Si substrate, hosting an integrated lumped-element LC resonator realized from a single layer of Nb–Ti–N and a Pt–C tip deposited at the cantilever apex. (d) Experimental setup. The scanner box is suspended from the 10 mK stage with soft springs. Folded copper braids provide thermalization and additional mechanical damping. The digital synthesizer and analyzer (Presto platform by Intermodulation Products AB) provides the DC output to control the scanner, the radio-frequency drive for the piezo shaker, and the microwave multifrequency lock-in modulation/demodulation.

The advantages of cavity optomechanical detection for AFM sensors were already showcased by Liu et al. [8], who implemented a whispering gallery mode toroidal optical cavity coupled through its evanescent field to a doubly clamped beam with a tip in the middle. We replace the optical cavity with a superconducting microwave resonant circuit, which is comparatively easy to fabricate and to integrate on an AFM cantilever with a tip at the free end. Figure 1a–c shows a scanning electron micrograph of the probe. The triangular cantilever is a Si–N plate released from a Si substrate. The microwave superconducting lumped-element resonant circuit consists of an interdigital capacitor in series with a meandering nanowire inductor, both fabricated from a single layer of Nb–Ti–N deposited on the Si–N. The nanowire meanders across the clamping edge of the plate, giving rise to a modulation of its kinetic inductance due to surface strain generated by cantilever bending. A Pt–C tip with curvature radius below 10 nm at its apex is formed at the free end of the cantilever through a series of electron-beam-assisted depositions.

Figure 1d shows a schematic of the experimental setup implementing a standard low-noise cryogenic microwave reflection measurement of the sensor, which is mounted in a prototype AFM suspended with three springs from the 10 mK plate of the DR. The microscope consists of a metallic box that supports the scanner and a printed-circuit board to which the sensor is mounted. This custom board also hosts a piezoelectric inertial actuator (shaker), which drives the cantilever oscillation. A test surface is mounted on top of the open-loop scanner stack consisting of a *z*-positioner for coarse approach and an open-loop *xyz*-scanner for fine positioning (Attocube anpz 102/LT/HV, ansxyz 100/LT/HV). Twisted pairs and coaxial cables are coiled to make springy electrical connections to the AFM. Thermal anchoring of the various parts is achieved by copper braids with multiple zig-zag folds. At room temperature, the resonance frequency of the AFM’s suspension system is roughly 1 Hz, with a quality factor of roughly 2.

Mechanical oscillation of the tip causes phase modulation of the reflected microwave pump, detected as motional sidebands in the signal spectrum. Measuring the microwave response at a sideband, the detection responsivity α [V/m] can be expressed as


[5]

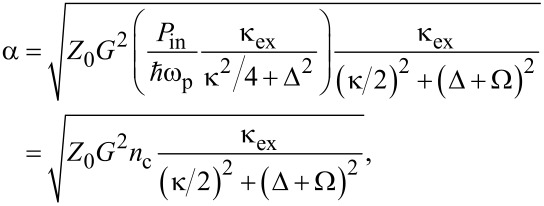



where *G* = ∂ω_0_/∂ζ [Hz/m] is the electromechanical coupling coefficient, κ_ex_ the external loss rate and κ the total loss rate of the microwave resonance, and *Z*_0_ is the characteristic impedance of the transmission line. The term in the first parenthesis is the circulating power or energy stored in the cavity, expressed as the intracavity photon number *n*_c_. We control *n*_c_ through the drive power of the pump *P*_in_ and the detuning from the cavity resonance frequency Δ = ω_p_ − ω_0_. In principle, arbitrarily large responsivity α is achieved for arbitrary weak coupling *G* by increasing *P*_in_. In practice, nonlinear effects emerge at large *n*_c_, such as heating (nonlinear loss) and resonance-frequency shift (Kerr nonlinearity), which cause the linear response picture of cavity optomechanics to break down.

Detection noise in the cavity optomechanical scheme is determined by quantum fluctuations of *n*_c_ or photon shot noise. Using Equation 5 for the responsivity α, we can convert this detection noise to an equivalent mechanical displacement noise or imprecision noise, inversely proportional to *n*_c_:


[6] $$S_{\zeta \zeta }^{\text{imp}}\left(\Omega \right)=\frac{\zeta_{\text{zpf}}^{2}}{4g_{0}^{2}n_{\text{c}}}\left(\frac{\kappa^{2}/4+{\left(\Omega +\Delta \right)}^{2}}{\kappa_{\text{ex}}}\right).$$

Here, we introduce the single photon coupling rate *g*_0_ = *G*ζ_zpf_, corresponding to the microwave resonance frequency shift associated with the quantum zero-point motion of the mechanical mode 
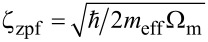
. The cavity optomechanical measurement also introduces a fluctuating radiation pressure force on the test mass or back-action noise, proportional to *n*_c_ [22–23]:


[7]

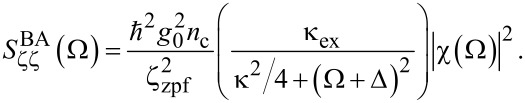



When the microwave pump frequency is detuned by Δ = −Ω_m_, and the mechanical mode is measured on resonance Ω = Ω_m_ where 
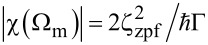
, detector noise is minimized when 
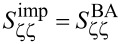
, where


[8] $$n_{\text{c}}=n_{\text{c}}^{\text{SQL}}=\frac{\Gamma }{4g_{0}^{2}}\frac{\kappa^{2}/4}{\kappa_{\text{ex}}}.$$

If the mechanical oscillator is cooled to a very low temperature, so that it is in its quantum ground state, a measurement at 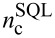 gives 
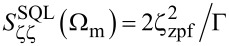
, the so-called standard quantum limit (SQL). In an actual experiment, components after the cavity in the measurement chain introduce additional imprecision noise, expressed as an added number of noise photons *n*_add_:


[9] $$S_{\zeta \zeta }^{\det}=S_{\zeta \zeta }^{\text{imp}}\left(1+2n_{\text{add}}\right)+S_{\zeta \zeta }^{\text{BA}}.$$

The added noise in our setup (Figure 1d) is determined by the cryogenic low-noise amplifier (LNA) mounted at the 4 K stage. We use Planck spectroscopy to calibrate our LNA, where we measure *n*_add_ ≈ 14 at 4.5 GHz [24–25]. The addition of a near quantum-limited amplifier (e.g., a Josephson parametric amplifier) as the first stage in the amplification chain, where *n*^add^ ≈ 1/2, is a commonly adopted solution to reduce detection noise [26].

### Sensor characterization

**Figure 2:**
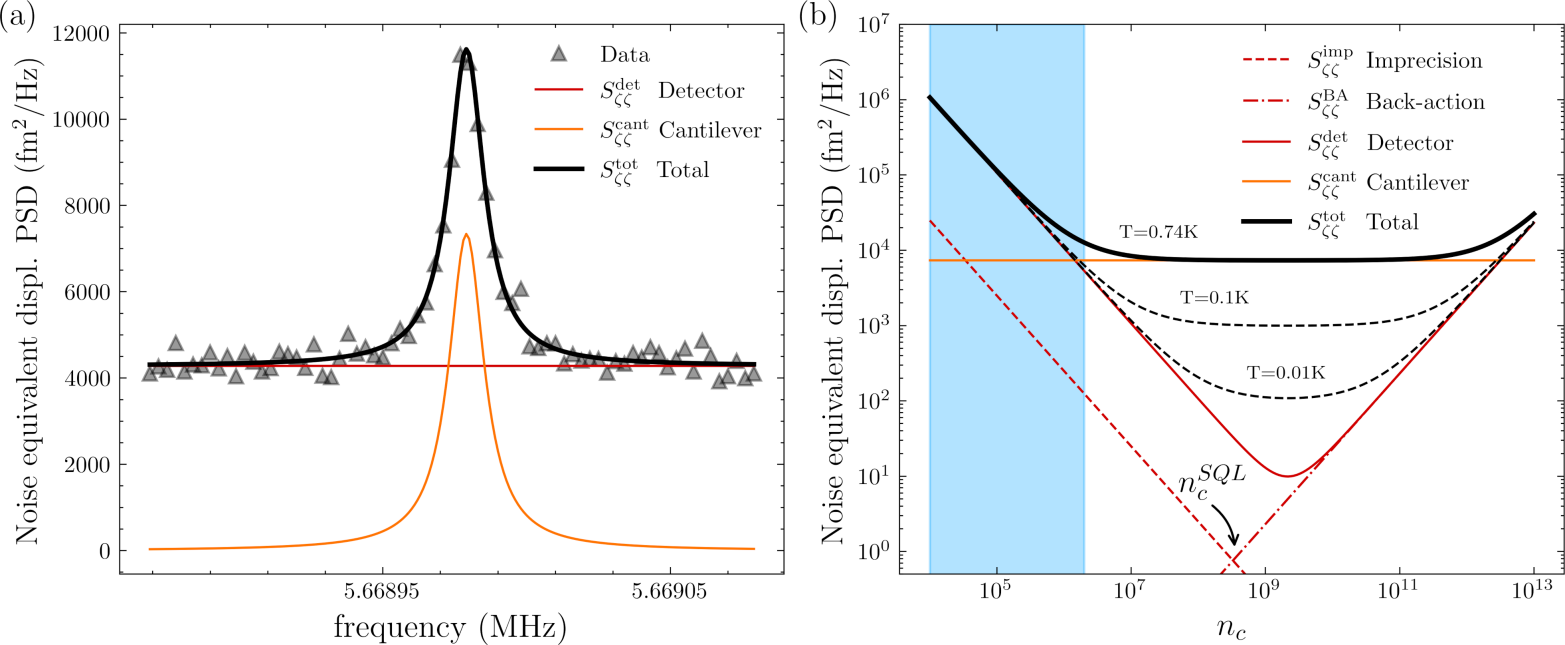
(a) Detector output noise equivalent displacement PSD and individual contributions. (b) Noise contributions evaluated at the mechanical resonance as a function of the average circulating photon number *n*_c_. The detector noise (solid red line) accounts for the intracavity ground state fluctuations (dashed red line), back action noise (dash-dot red line) and the added noise of the first stage of amplification. The horizontal line marks the fluctuations of the cantilever at its effective mode temperature. The solid and dashed black curves describe the total noise of the sensor for various cantilever mode temperatures.

Table 1 summarizes the device parameters for three different probes employed in this work, measured at 10 mK in free space far from the surface. We characterize the sensor by measuring the total noise of the undriven mechanical resonance, up-converted to the microwave spectrum. Figure 2a shows the data for probe 1, obtained by converting the measured upper motional sideband to an equivalent displacement PSD 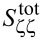 through Equation 5 with Δ = −Ω_m_ and *n*_c_ = 1.98 × 10^6^. Fitting Equation 2 to the measured data, we extract the detector (red) and cantilever (orange) contributions to the total noise. Through this fitting procedure we determine an effective temperature of the cantilever *T* = 0.74 K, which is substantially higher than the bath temperature *T*_bath_ = 10 mK. The discrepancy is most likely due to inertial actuation by noise from the piezo shaker and decoupling of the cantilever eigenmode from the thermal bath.

**Table 1:** Device parameters Ω_m_ and Γ are determined by fitting the measured mechanical resonance. ω_0_, κ and κ_ex_ are determined by fitting to the measured microwave resonance, while *m*_eff_ and *G* are determined from FEM simulations [18].

Probe	Ω_m_ (MHz)	Γ (Hz)	*m*_eff_ (pg)	ω_0_ (Ghz)	κ (MHz)	κ_ex_ (MHz)	G (kHz/nm)

1	5.669	15	47	4.27	3.38	3.04	24.92
2	4.963	5	61	4.64	1.03	0.64	24.56
3	5.881	45	44	4.40	4.54	4.04	28.98

Figure 2b shows various contributions to the noise of probe 1 as a function of *n*_c_. The light blue area marks the range of operation of this sensor, where Kerr nonlinear response of the superconducting resonant circuit [27] limits *n*_c_ *<* 2.0 × 10^6^, well below 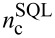 ≈ 1.4 × 10^8^.

The general requirement for achieving SQL is the same as for any dispersively coupled optomechanical system, that is, operating the cavity at the optimal intracavity photon number 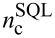 and maximizing the measurement efficiency (κ_ex_ ≈ κ, *n*_add_ ≈ 0). Operating the sensor near the optimal point requires 
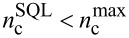
. For a given *g*_0_ and Γ we can decrease 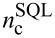 by reducing the external loss rate κ_ex_ and the total loss rate κ. We can control κ_ex_ through design of the resonator coupling to the readout transmission line [18]. Decreasing κ also requires improvement on the internal loss rate of the cavity κ_0_ = κ − κ_ex_. With typical parameters for our sensors, κ_ex_/2π = 1 MHz, Γ/2π = 10 Hz, and *g*_0_/2π = 0.14 Hz, we find a value of 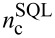 ≈ 31.8 × 10^6^ photons, which would require an improvement on κ_0_ by a factor of 15. Achieving this improvement is quite reasonable as our microwave resonators exhibit κ_0_ values far larger than the typical values reported in the literature [28–30]. The main limitation to the internal quality factor of our microwave resonators is the highly disordered low-stress Si–N layer, which causes significant dielectric losses [31].

As discussed above, the figure of merit of a displacement detector should be evaluated with respect to the fluctuations of the specific mechanical resonator that is measuring the force. In this regard, it is informative to define the crossover temperature T^*^, or effective mode temperature of the mechanical resonator, at which 
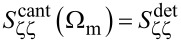
. When the mechanical mode temperature T > T^*^, the noise is dominated by mechanical displacement noise and the force measurement is at the thermal limit. When T < T^*^, the measurement is dominated by detector noise and improvements to displacement detection will improve force sensitivity.

Piezoelectric sensors with transimpedance amplifier detectors as reported by Giessibl et al. [32] exhibit 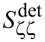 = 62 fm/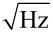 giving T^*^ = 10 K for tuning forks, and 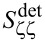 = 1.89 fm/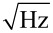, giving T^*^ = 21 K for length-extensional resonators. Optimized optical-beam deflection detectors as reported by Fukuma et al. [33] exhibit 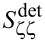 = 17 fm/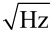, T^*^ = 1.2 K. For comparison, from measurements on probe 1 shown in Figure 2a, we obtain a lower crossover temperature T^*^ = 0.43 K, despite the fact that the sensor operates below its optimal point.

### AFM operation

The noise-to-noise ratio 
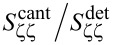
 = *T*/T^*^ provides a qualitative measure of the pixel acquisition rate, or measurement bandwidth Δ*f*, above which force sensitivity is degraded by detector noise. For FM-AFM this bandwidth is Δ*f* = Γ(*T*/T^*^), whereas AM-AFM requires Δ*f* ≤ Γ. Force sensitivity is, however, only one aspect of a high-performance AFM. Ultimately the image speed and resolution, both vertical and lateral, are influenced by many factors, namely, equilibrium tip–surface distance, oscillation amplitude of the driven cantilever, sharpness of the tip, and pickup of environmental vibrations and noise, such as noise from the actuator driving the cantilever oscillation as well as feedback noise and sample topography.

We perform initial positioning of the microscope by driving the coarse *z*-positioner with the Attocube ANC-300 control unit that provides the 60 V saw-tooth pulses to the piezo actuators. The stick–slip motion of the actuator under high-voltage saw-tooth pulses (≈50 nm/step) produces significant heating at the 10 mK stage. Under continuous operation, the heating power is of the order of 1 µW for an approach speed of 1 µm/min. This temperature change during coarse approach causes drift in the cantilever’s resonance frequency, requiring a couple of minutes to restore a stable configuration. Therefore, during coarse approach we monitor only the microwave resonance.

**Figure 3:**
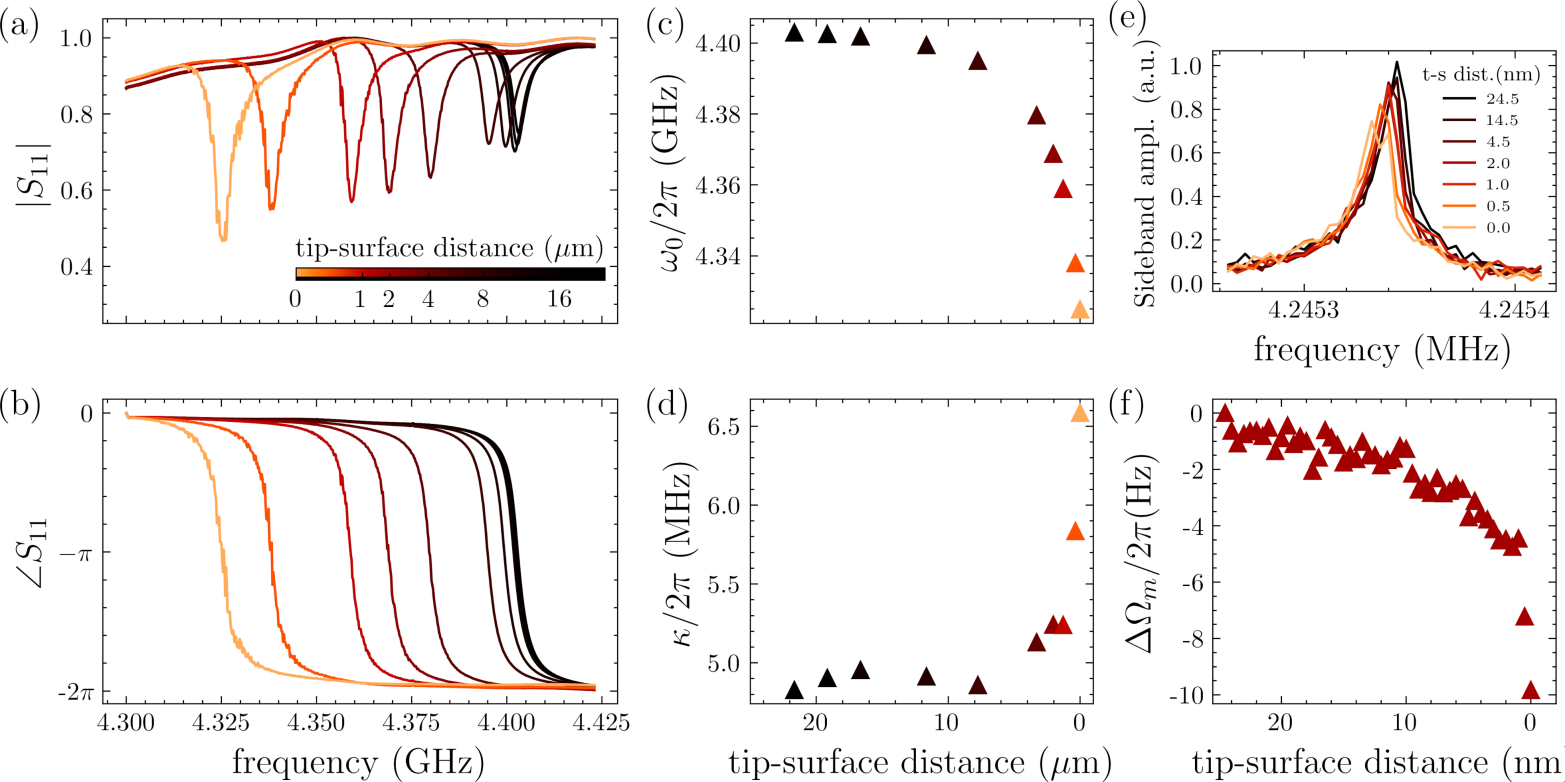
(a) Magnitude and (b) phase of the microwave resonator’s reflection coefficient *S*_11_ measured during coarse approach. Proximity to the surface causes (c) a non-negligible shift in the microwave resonance frequency and (d) an increase in the total loss rate. (e) Effect of force gradient on the mechanical susceptibility of the cantilever close to the surface. (f) Mechanical resonance frequency shift as a function of the tip–surface distance.

When approaching the sample surface, we observe a shift (*<*2%) of the microwave resonance frequency to lower frequency and a slight increase of the internal losses, as shown in Figure 3a–d. This redshift suggests that proximity to the surface introduces a lossy capacitive load to the microwave resonant circuit. While slightly degrading the performance of the displacement detector, this effect has the advantage of providing an easily measurable indication of probe–surface separation during coarse approach. For the final stage of the approach, we drive the *z*-scanner with a DC voltage (100 nm/V) while monitoring both the microwave and cantilever resonances. Figure 3e,f shows the measured frequency shift as a function of the distance, demonstrating force-gradient sensing by the KIMEC AFM probe.

We tested the AFM by imaging a nanofabricated calibration grating over a 1 µm × 1 µm scan area with a resolution of 100 pixels/line. In order to track the surface topography while scanning, we developed a proportional-integral-derivative (PID) control firmware running on the embedded real-time processing unit of the microwave multifrequency lock-in amplifier (Presto by Intermodulation Products AB). The feedback system must be able to follow rapid changes in topography while avoiding feedback oscillation. Depending on the mode of operation, AM-AFM or FM-AFM, the PID setpoint and error signal are determined, respectively, from the amplitude or phase of the motional sideband, which is the up-converted cantilever oscillation.

The PID control is developed in the Rust programming language and features low-pass filtering of the derivative action, as well as clamping of the integrator state to prevent integral windup and of the output control resulting in damage to the AFM probe. The code is released as open source under the MIT license and is available online in a repository hosted on GitHub [34].

**Figure 4:**
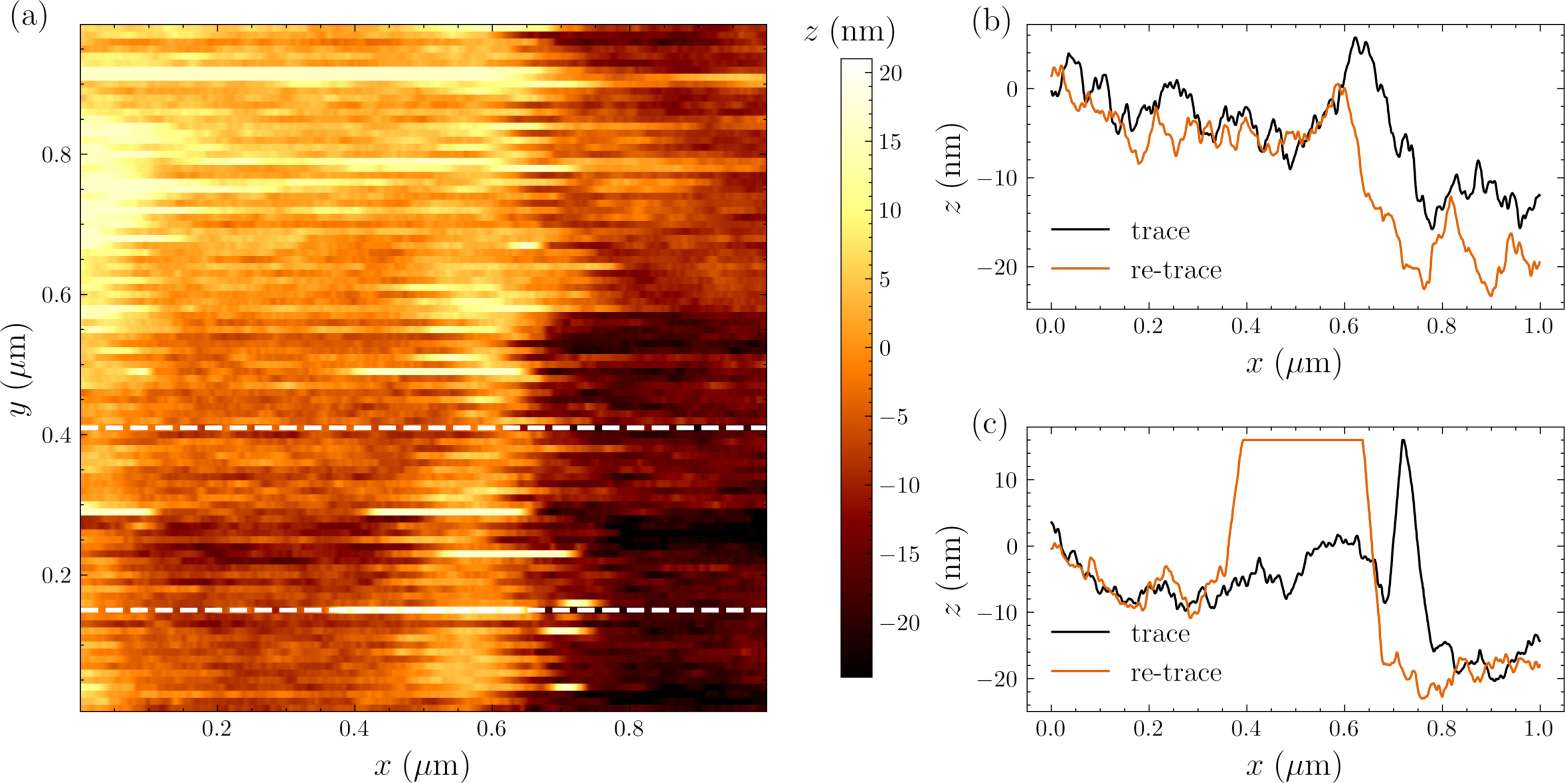
(a) AM-AFM imaging of the calibration grating over a 1 µm × 1 µm scan area containing a single vertical stripe with a 10 nm step height. The dashed lines in the image mark the location of the scan lines in (b) and (c).

We operate probe 2 in AM-AFM mode by driving the cantilever slightly above resonance Ω = Ω_m_ + Γ/2. The PID loop controls the *z*-extension of the scanner to keep oscillation amplitude constant at about 200 pm. The blue-detuned cantilever drive ensures that the PI controller retracts the *z*-scanner in the event of a sudden drop in the measured signal, thus avoiding crashing the probe into the surface. The image in Figure 4a is acquired with a measurement bandwidth of Δ*f* = 1.5 Hz *<* Γ, corresponding to a scan rate of roughly 67 s/line. The choice of the integral gain is crucial for optimal surface tracking. Too large values of integral gain result in an overly responsive feedback that introduces noise on the topographic image and feedback oscillations. Too low values can lead to instability or loss of surface tracking with abrupt changes in topography. The latter occurred during the scan; in multiple instances, the feedback temporarily loses track of the surface immediately after the tip encounters a step. The single scan line on the fast axis (i.e., *x*-axis) shown in Figure 4b,c clearly shows different behavior of trace and retrace scan directions.

**Figure 5:**
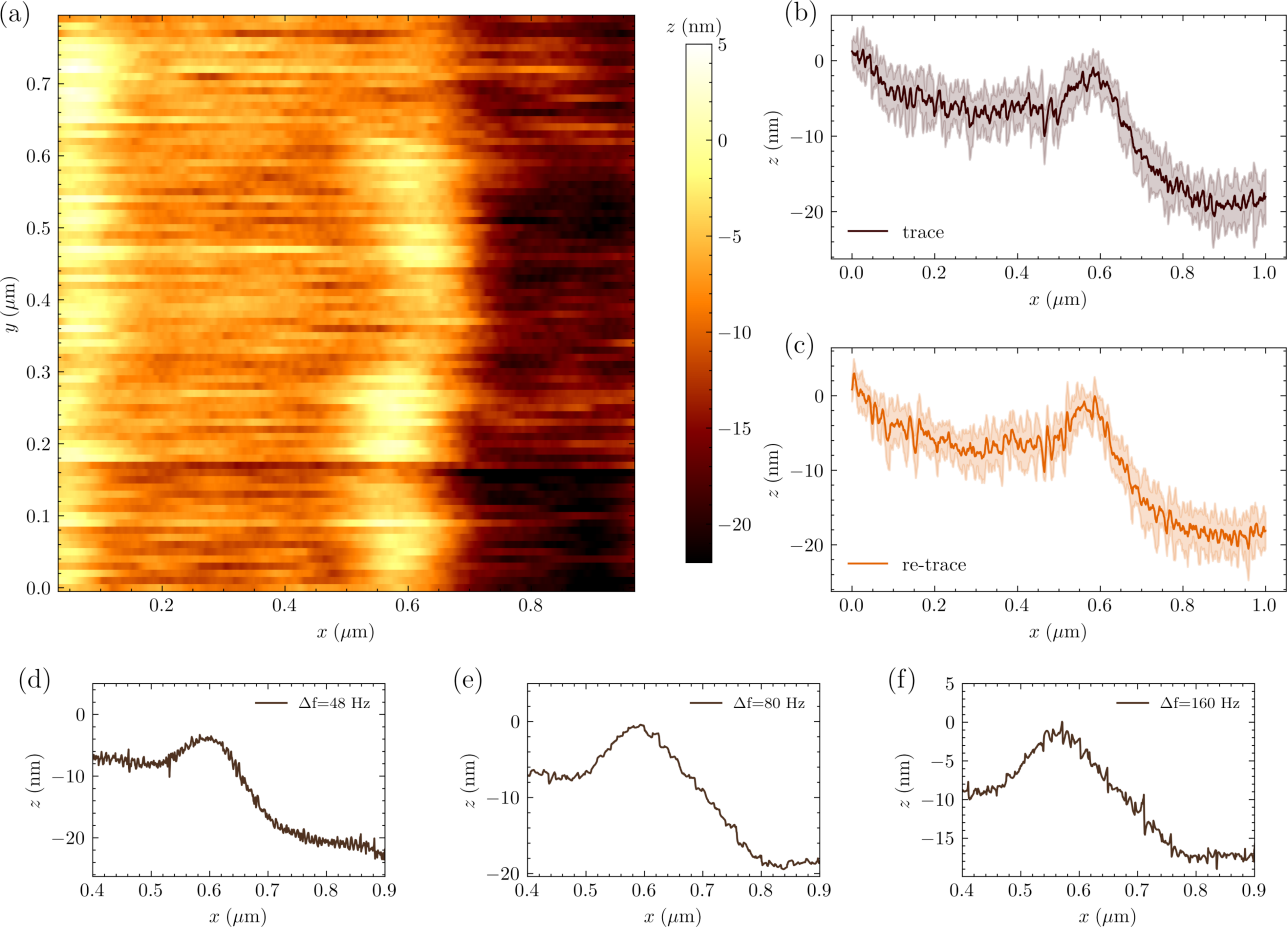
(a) FM-AFM imaging (trace) of the calibration grating over a 1 µm × 0.8 µm scan area. (b, c) Average and standard deviation over ten consecutive acquisitions of a single scan line at constant *y*-position in both trace (b) and retrace (c) scan direction. (d–f) Single scan line measured with different pixel acquisition rates Δ*f*.

We achieved faster imaging and improved stability with FM-AFM as shown in Figure 5. FM-AFM requires phase-sensitive detection of tip displacement using a scheme described in detail in earlier publications [35,17]. The cantilever is now driven on resonance at the same ≃200 pm oscillation amplitude while the microwave resonator is driven with two pump tones of equal intensity and symmetrically detuned with Δ = ±Ω_m_. The lower and upper motional sidebands from the two pumps interfere at ω_0_, resulting in a microwave response amplitude that depends on the phase of the mechanical motion [36–37]. The feedback controls the *z*-extension to maintain a constant microwave response amplitude, or constant mechanical response phase. The tracking of the surface is more consistent using this FM-AFM feedback scheme as shown in Figure 5b,c by the trace and retrace signals. Figure 5d–f shows the effect of the measurement bandwidth on a single scan line.

**Figure 6:**
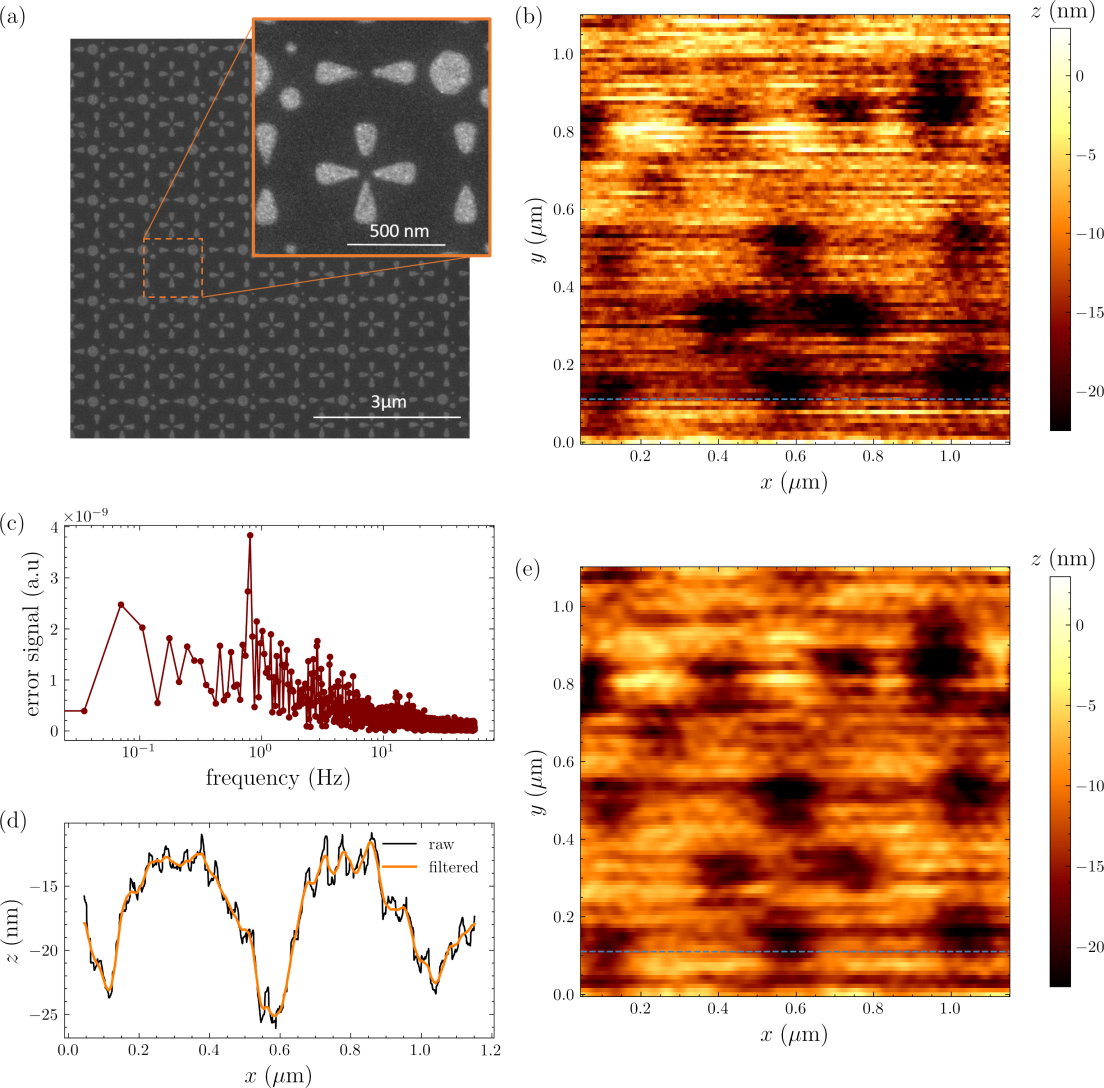
(a) SEM image of the second test sample. The zoomed inset highlights the area imaged with FM-AFM in (b). (c) Fast-Fourier transform of the error signal for a single scan line. The *x*-axis of the plot is converted to frequency through the pixel acquisition rate Δ*f* = 112 Hz. (d) Single scan line showing the rippling effect caused by pulse-tube vibrations. The orange curve is obtained by applying a low-pass filter with cutoff frequency 0.75 Hz. (e) Effect of filtering and smoothing on the acquired image.

Finally, we tested the lateral resolution of our microscope by imaging a less trivial structure. We fabricated a second test sample by etching a pattern from the top Ti layer of a Ti(10 nm)/Au(5 nm)/Ti(5 nm) film deposited on a silicon substrate. Each unit cell in the pattern contains different shapes as shown in the SEM image in Figure 6a. The tapered arms of crosses and ribbons shrink to reach a nominal minimum feature size of 20 nm. Figure 6b shows a FM-AFM image of the 1 µm × 1 µm scan area corresponding to the inset of Figure 6a acquired using probe 3.

Although the various structures can be identified, several scanning artifacts appear in the image. In this particular experiment the probe was especially sensitive to pulse-tube vibrations. Figure 6c shows the fast-Fourier transform (FFT) of the error signal for a single horizontal (fast axis) line scan. The component at 0.8 Hz and its higher harmonics coincide with periodicity of the pulse-tube, resulting in ripples in the height image (feedback signal) as shown in Figure 6d. In this image we also see distortion and misalignment between consecutive scan lines due to hysteresis and drift in the open loop *x*–*y* scanners. These problems are more pronounced when attempting to resolve smaller features, and image processing techniques such as filtering and smoothing are not particularly effective in enhancing resolution, as shown by Figure 6e.

## Conclusion

We have demonstrated ultralow-temperature AFM operation with a kinetic-inductive electromechanical (KIMEC) force sensor. In comparison with state-of-the-art low-temperature AFM, these initial AFM images are not of particularly high resolution. However, our AFM was built into a standard pulse-tube dilution refrigerator, which was not designed for preparation of pristine surfaces, nor was any attempt made to sharpen the tip in situ.

Our analysis shows that the KIMEC principle of detecting cantilever deflection, as it is implemented in these sensors, is capable of operating at the thermal limit of force sensitivity down to a temperature of 0.43 K. This is a significant improvement over piezoelectric transduction methods with much lower frequency commonly used for low-temperature AFM, which can work at the thermal limit down to some 10 K. We estimate that, with reasonable improvements on the current KIMEC sensor design, this detection principle will be capable of operating at the fundamental limit of minimum added noise. Achieving this performance limit is a prerequisite for improved force sensitivity with ultralow temperature and ultralow damping mechanical modes. Alternatively, the lower noise of the KIMEC detection principle allows for AFM imaging with increased pixel acquisition rate (measurement bandwidth) without degrading force sensitivity.

## Data Availability

Data generated and analyzed during this study is openly available in Zenodo at https://doi.org/10.5281/zenodo.15744435.
